# Decreased thalamo-cortico connectivity during an implicit sequence motor learning task and 7 days escitalopram intake

**DOI:** 10.1038/s41598-021-94009-7

**Published:** 2021-07-23

**Authors:** Eóin N. Molloy, Rachel G. Zsido, Fabian A. Piecha, Nathalie Beinhölzl, Ulrike Scharrer, Gergana Zheleva, Ralf Regenthal, Bernhard Sehm, Vadim V. Nikulin, Harald E. Möller, Arno Villringer, Julia Sacher, Karsten Mueller

**Affiliations:** 1 Emotion and Neuroimaging Lab, Max Planck Institute for Human Cognitive and Brain Sciences, Leipzig, Germany; 2 Department of Neurology, Max Planck Institute for Human Cognitive and Brain Sciences, Stephanstr. 1A, 04103 Leipzig, Germany; 3 International Max Planck Research School NeuroCom, Max Planck Institute for Human Cognitive and Brain Sciences, Leipzig, Germany; 4 Max Planck School of Cognition, Leipzig, Germany; 5 Division of Clinical Pharmacology, Rudolf-Boehm-Institute of Pharmacology and Toxicology, Leipzig University, Leipzig, Germany; 6 Centre for Cognition and Decision Making, Institute for Cognitive Neuroscience, National Research University Higher School of Economics, Moscow, Russia; 7 Nuclear Magnetic Resonance Methods and Development Group, Max Planck Institute for Human Cognitive and Brain Sciences, Leipzig, Germany; 8 MindBrainBody Institute, Berlin School of Mind and Brain, Charité-Berlin University of Medicine and Humboldt University Berlin, Berlin, Germany; 9 Clinic of Cognitive Neurology, University Hospital Leipzig, Leipzig, Germany; 10 Department of Neurology, University Hospital Halle (Saale), Halle, Germany

**Keywords:** Neuroscience, Learning and memory, Motor control, Neurochemistry, Pharmacology

## Abstract

Evidence suggests that selective serotonin reuptake inhibitors (SSRIs) reorganize neural networks via a transient window of neuroplasticity. While previous findings support an effect of SSRIs on intrinsic functional connectivity, little is known regarding the influence of SSRI-administration on connectivity during sequence motor learning. To investigate this, we administered 20 mg escitalopram or placebo for 1-week to 60 healthy female participants undergoing concurrent functional magnetic resonance imaging and sequence motor training in a double-blind randomized controlled design. We assessed task-modulated functional connectivity with a psycho-physiological interaction (PPI) analysis in the thalamus, putamen, cerebellum, dorsal premotor, primary motor, supplementary motor, and dorsolateral prefrontal cortices. Comparing an implicit sequence learning condition to a control learning condition, we observed decreased connectivity between the thalamus and bilateral motor regions after 7 days of escitalopram intake. Additionally, we observed a negative correlation between plasma escitalopram levels and PPI connectivity changes, with higher escitalopram levels being associated with greater thalamo-cortico decreases. Our results suggest that escitalopram enhances network-level processing efficiency during sequence motor learning, despite no changes in behaviour. Future studies in more diverse samples, however, with quantitative imaging of neurochemical markers of excitation and inhibition, are necessary to further assess neural responses to escitalopram.

## Introduction

Motor learning is an anatomically diverse process, drawing on contributions from the primary, supplementary, premotor, prefrontal cortices, and cerebellum^1,2^. Interactions between these regions are dynamic, and both functional and structural motor connectivity can be strengthened by practice^3^ or altered by disease^4^. Previous conceptual models propose that selective serotonin reuptake inhibitors (SSRIs), anti-depressant and anxiolytic medications that increase extracellular serotonin^5^, act by reorganizing neural networks via a transient window of neuroplasticity^6,7^. Beyond the clear relevance for psychiatric disorders, this hypothesis has also gained traction in clinical models of post-stroke motor rehabilitation^8^. Despite this, recent clinical trials^9,10^ investigating the role of SSRIs in motor recovery in stroke did not assess neural responses to SSRI administration, and previous studies in health are largely limited to assessments of neural activity. Considering that functional connectivity can provide a valuable proxy for investigating pharmacological interventions with complex neuromodulatory action, assessing the influence of SSRIs on motor network connectivity in healthy volunteers may provide an important first step in understanding this effect.

Previous studies support a modulatory role of SSRIs in human motor function. For example, acute administration of paroxetine has been shown to enhance general motor performance on the Nine Peg Hole and Moede Dexteritymeter tasks^11^ while a single dose of fluoxetine has been associated with modulation of cortical excitability during a finger tapping task^12^. Performance of spatially motivated dexterity tasks has also been shown to correlate with decreased functional brain responses following chronic administration of paroxetine^13^, while our previous work shows that 1-week of escitalopram intake reduces premotor cortex response during sequential motor learning, despite comparable behavioural performance to a placebo group^14^. Preliminary evidence for a network-level modulation effect of SSRIs has also been reported with a single dose of escitalopram altering connectivity between sensorimotor regions during a pilot study of older adults performing a simple finger movement task^15^. Task-independent fMRI studies have also supported a connectome-wide effect of SSRIs. For example, an intravenous dose of 10 mg citalopram increased prefrontal connectivity in healthy volunteers^16^ while studies with 1-week administration of citalopram report decreases in prefrontal connectivity in healthy volunteers^17^. Moreover, acute intake of escitalopram has been associated with bi-directional changes in resting state brain connectivity, with decreased connectivity in cortical regions and increases in subcortical brain structures^18^. Consequently, several lines of evidence suggest that SSRI-administration can exhibit widespread neuromodulatory effects on intrinsic functional brain connectivity in healthy humans.

While these studies have provided promising insights regarding motor performance in terms of behavioral outcomes, neural activation, or task-independent fMRI, the effect of SSRI-administration on functional connectivity *during* motor learning is still largely unknown. One area of interest is *sequence* motor learning, a domain which, despite heterogenous findings in stroke patients^19,20^, is generally thought to be impaired^21^. Given the central role of sequence skill movement in the performance of everyday tasks, assessing the effects of SSRIs on the functional connectivity patterns underlying normal sequence motor learning thus represents a critical target for preclinical stroke research in human participants. Moreover, assessing how SSRIs exert this effect can be readily conducted with psycho-physiological interaction (PPI); a method that, in contrast to the univariate *voxel-by-voxel* approach of fMRI measures of brain activity, assesses the *relationship between voxels*. This bivariate approach enables researchers to probe undirected functional connectivity responses during task conditions and to model the integration of distinct brain regions during network-level task processing^22–24^. By assessing functional connectivity in the presence of a motor paradigm, PPI allows for the investigation of SSRI-modulated functional connectivity during sequence motor learning.

The present study thus aims to address the question of whether SSRI-intake alters PPI connectivity across 5 days of implicit sequence motor learning. To do this, we have combined 1-week administration of 20 mg escitalopram, a common SSRI with a relatively fast onset of action^25^, with fMRI and a variant of the sequential pinch force task (SPFT) to sixty healthy female participants in a double-blind placebo-controlled design. To test our hypothesis, we selected seven regions of the motor network known to be involved in sequence motor learning behavior, planning, and motor network connectivity: the left hemisphere primary motor cortex (M1)^26,27^, supplementary motor area (SMA)^28,29^, putamen^30^, thalamus^31,32^, cerebellum^29,30^, and dorsal premotor cortex (dPMC)^30^. Based on previous findings in healthy right-handed participants undergoing sequence motor training^26^, we also selected the right dorsolateral prefrontal cortex (dlPFC)^33^. We employed a PPI analysis to investigate the effects of escitalopram on functional connectivity associated with sequence motor learning (hereafter referred to as the *PPI Learning contrast*), and, in a second analysis, generalized motor performance (hereafter referred to as the *PPI Motor Contrast*). With each seed region having been implemented in human motor learning and plasticity, we hypothesized that, in the absence of an effect on behavioral performance^14^, 1-week escitalopram administration would lead to a significant change in PPI functional connectivity with respect to both sequence motor and generalized motor task conditions.

## Results

### Screening variables

Group demographic comparisons show no significant differences for age, BMI, or downregulated hormonal profile (Table 1). Plasma escitalopram levels are within the expected range (single dose = 20 ± 5 ng/ml, steady state = 46 ± 11 ng/ml)^34^.

**Table 1:** Results from independent sample *t*-tests assessing potential group differences for participant age, body mass index (BMI), Follicle stimulating hormone (FSH) levels, Luteinizing hormone (LH) levels. Mean ± Standard Deviation (M ± SD).

Demographic	Placebo (M ± SD)	Escitalopram (M ± SD)	*t* value	*p* value
Age (years)	23 ± 4	24 ± 3	1.27	0.20
BMI (kg/m^2^)	21 ± 1.2	21.8 ± 2	− 1.3	0.20
FSH (IU/l)	2 ± 3	3 ± 3	− 1.2	0.24
LH (IU/l)	1.4 ± 2	2 ± 2.6	1.0	0.32

### Behavioral performance

All participants completed 5 days training on the SPFT, including a sequence learning condition (Sequence Learning) and a simpler motor learning condition (Simple Learning). A Rest condition with no behavioral input interspersed performance of the Sequence Learning and Simple Learning conditions and was retained for use in fMRI model specification. Comparison of motor performance between the Sequence Learning and Simple Learning conditions yielded a significant effect, with performance differing between conditions (see Supplemental Table 1; Supplemental Fig S3). Despite significant improvements in performance for all participants over time, we did not observe an effect of escitalopram on behavioral measures of sequence-specific motor learning. Interpretation of these findings are reported elsewhere^14^.

### Group comparisons of functional connectivity changes over time

**Figure 1:**
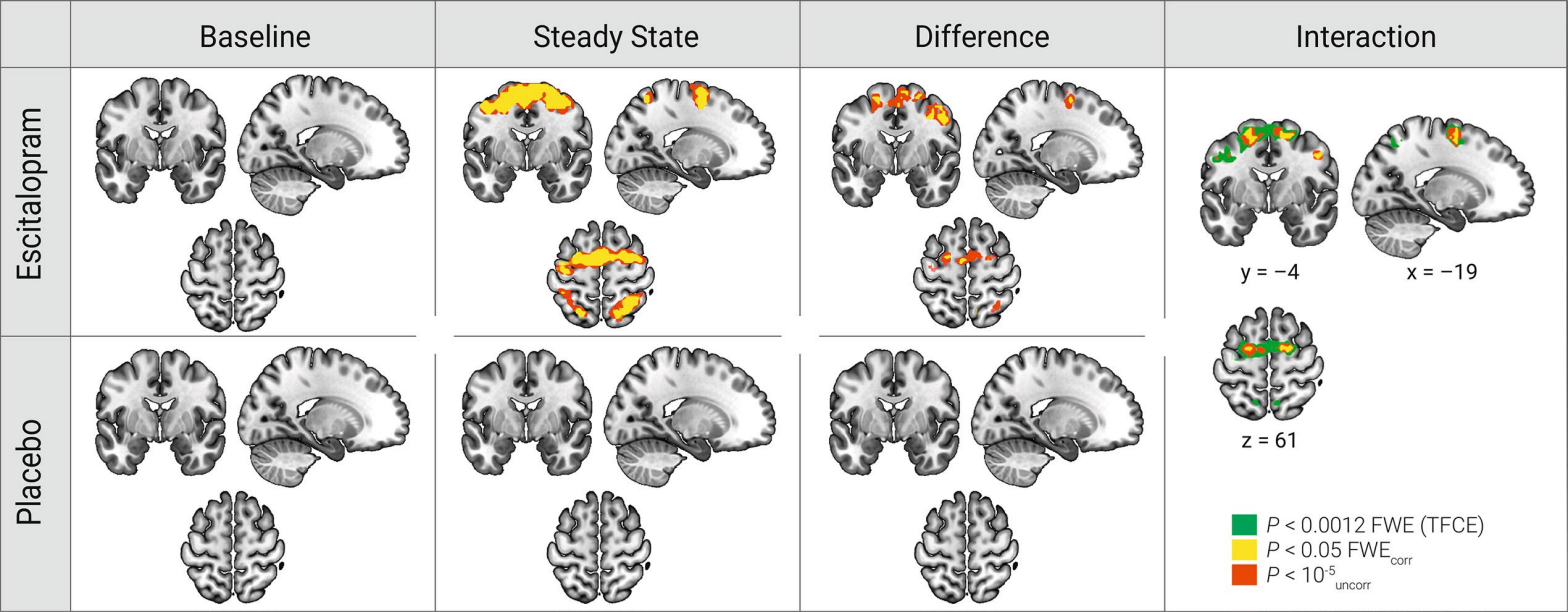
Orthogonal brain slices of PPI Learning contrast showing a thalamo-cortico connectivity decrease in response to escitalopram. Significant brain connectivity decrease in the PPI Learning contrast were observed after 7 days of 20 mg escitalopram administration (*Difference*—top row). In contrast, no task-related connectivity changes were observed within placebo (*Difference*—bottom row). A 2 × 2 interaction analysis revealed a significant group difference over time (*Interaction*). Results were obtained with *p* < 0.05 with FWE correction at the voxel level (yellow). Also shown is an uncorrected cluster forming threshold of *p* < 10^–5^ with an extent of ≥ 20 voxels (red), and results from a non-parametric analysis of the interaction contrast (green) corrected for multiple comparisons (*p* < 0.0012).

For the thalamus seed region, we observed a significant group × time interaction when comparing the PPI Learning contrast (i.e. the difference in connectivity between the Sequence and Simple Learning conditions) between steady state and baseline, with a significant decrease in connectivity within the escitalopram group, not mirrored in the placebo group. Specifically, we observed a decrease of the PPI Learning contrast between the thalamus and cortical brain regions of the motor system including bi-hemispheric primary and premotor regions (Table 2; Fig. 1). Non-parametric permutation tests replicated this finding with a significant decrease of PPI thalamo-cortico connectivity in bilateral premotor and parietal regions (Table 3) within the escitalopram group. No significant interaction was observed when comparing single dose to baseline or single dose to steady state.

**Table 2:** 2 × 2 analyses comparing group changes in the PPI Learning contrast over time show a significant decrease in connectivity from the thalamus seed region to the bilateral primary and premotor regions in response to escitalopram (*Interaction*).

Region	Peak *p*(FWE_corr_)	Voxels	*t*-value	*z-*value	MNI (x,y,z)
**Interaction**					
R. primary motor	0.003	13	6.05	5.30	48, − 1, 41
R. premotor	0.003	17	5.97	5.25	18, − 4, 59
L. rolandic operculum	0.005	6	5.83	5.15	− 45, 5, 14
L. premotor	0.007	30	5.74	5.09	− 18, − 4, 56
			5.57	4.97	− 6, − 7, 65
			5.56	4.95	− 18, − 4, 65
L. primary motor	0.007	13	5.73	5.08	− 39, 2, 38
			5.15	4.65	− 48, 2, 38
L. middle occipital gyrus	0.014	10	5.53	4.94	− 27, − 70, 26
**Post-hoc**					
R. primary motor	0.001	54	6.24	5.44	51, − 1, 35
					36, − 4, 44
					42, − 4, 53
R. superior parietal lobule	0.003	24	5.98	5.25	27, − 58, 56
L. posterior medial frontal/supp motor	0.006	25	5.80	5.13	− 9, − 7, 56
R. supramarginal gyrus	0.008	7	5.71	5.07	63, − 31, 41
L. inferior parietal lobule	0.010	7	5.64	5.01	− 45, − 25, 38
R. superior frontal gyrus	0.010	8	5.63	5.01	24, − 4, 65
L. primary motor	0.010	9	5.63	5.01	− 39, − 13, 56
L. inferior parietal lobule	0.010	5	5.62	5.00	− 30, − 46, 47
L. premotor	0.016	8	5.49	4.90	− 18, − 4, 56
R. supramarginal gyrus	0.021	6	5.41	4.84	42, − 31, 38
R. posterior/medial frontal gyrus	0.021	6	5.39	4.84	12, − 4, 68

Post-hoc analyses reveal significant decreases in PPI connectivity between baseline and steady state within the thalamus seed region of the escitalopram group (*Post*-*hoc*). Results shown at a *p* < 0.05 Family-Wise Error corrected (FWE_corr_) on the whole brain voxel level. Significant results with < 5 voxel extent not shown.

*L* left, *R* right, *peak MNI* Montréal Neurological Institute coordinates.

**Table 3:** Non-parametric TFCE results from significant 2 × 2 group by time interaction estimated with 5000 permutations for the thalamus seed region.

Region	Peak *p*(FWE_corr_)	Voxels	TFCE	MNI (x,y,z)
**Interaction**				
L/R. premotor	< 0.001	589	1931.53	− 18, − 4, 56
	< 0.001		1923.62	− 18, − 4, 65
	< 0.001		1915.94	18, − 4, 59
L. inferior parietal lobule	0.001	167	1770.59	− 30, -49, 47
	0.001		1743.86	− 30, − 58, 53
	0.001		1733.82	− 45, -28, 38
L. rolandic operculum	0.001	36	1729.63	− 45, 5, 14
L. precuneus	0.001	48	1690.04	− 9, − 73, 50
			1676.65	− 18, − 67, 53
			1658.24	− 15 − 61, 47
R. precuneus	0.001	20	1653.64	9, − 61, 62
				6, − 67, 53
L. middle occipital gyrus	0.001	9	1632.08	− 27, − 70, 26
R. superior parietal lobule	0.001	1	1613.63	27, − 58, 56

Results show a significant decrease in the PPI Learning contrast from the thalamus to the bilateral premotor, parietal, and occipital regions. Results shown at a *p* < 0.0012 family-wise error corrected (FWE_corr_) on the whole brain voxel level.

*L* left, *R* right, *peak MNI* Montréal Neurological Institute coordinates, *TFCE* threshold Free Cluster Enhancement.

Analyses of the PPI Learning contrast with the other seed-regions within the M1, SMA, cerebellum, putamen, dPMC, and dlPFC, revealed no significant group × time interaction in our statistical approach using non-parametric permutation tests including correction for multiple comparisons. As a result, no post-hoc tests were performed for these seed regions. For the PPI Motor contrast (the comparison between the combined Sequence and Simple Learning conditions to the Rest condition), no significant group × time interaction was observed for any seed-region. Thus, no post-hoc nor non-parametric analyses were performed for this contrast.

### Post-hoc tests for the PPI Learning contrast with the thalamus seed region

Post-hoc paired comparisons for the escitalopram group yielded a significant change in the PPI Learning contrast from baseline to steady state between the thalamus and bilateral primary motor and parietal regions (Table 2—*Post-hoc*; Fig. 1—top row *Difference*). A paired comparison for the placebo group yielded no significant change (Fig. 1—bottom row *Difference*). Finally, an independent samples *t*-test at baseline, yields no significant group differences.

### Estimates of effect size of the PPI Learning contrast

**Figure 2:**
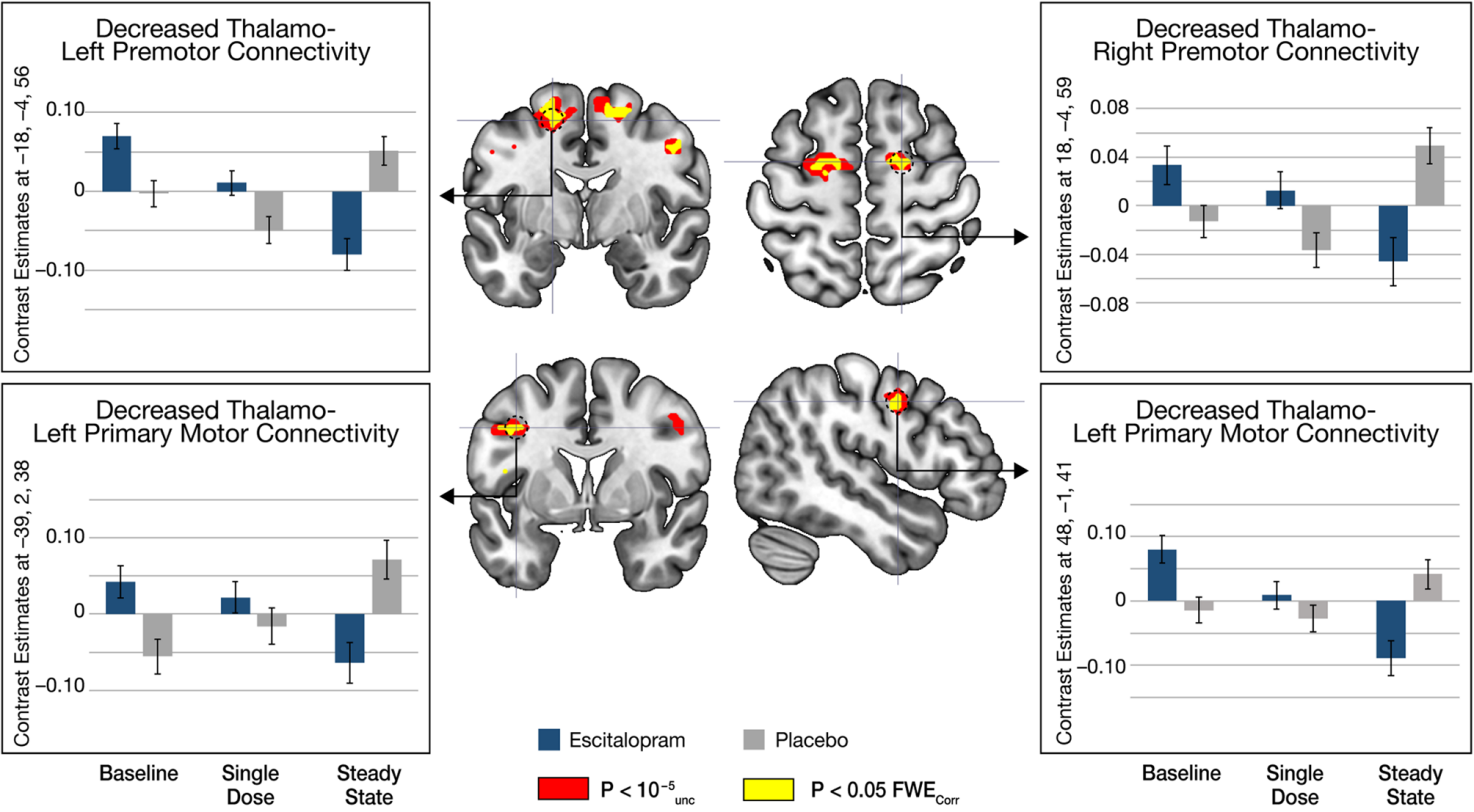
Contrast estimates from motor regions showing a gradual decreases in the thalamo-cortico PPI Learning contrast from baseline to steady state. Contrast estimates extracted from the escitalopram group motor regions at each time point show a gradual decrease in the PPI Learning contrast over the course of the administration week. Orthogonal brain slices show regions significant in a 2 × 2 flexible factorial analysis comparing groups from baseline to steady state. Results are shown at both a *p* < 10^–5^ cluster corrected threshold (red) and *p* < 0.05 with FWE correction at the voxel level (yellow).

Contrast estimates for the PPI Learning contrast in the escitalopram group show a gradual decrease in intensity associated with the Sequence Learning condition over time. Specifically, results show a linear pattern of decrease in the PPI Learning contrast for the sequence motor Learning condition from baseline to steady state in each motor region resulting from our group × time interaction analysis. Contrast estimates for the placebo group indicate an increase in thalamo-cortico connectivity from baseline to steady state, however, these changes were not significant in post-hoc paired comparisons within the placebo group (Fig. 2).

### Associations between thalamo-cortico connectivity changes and escitalopram kinetics

**Figure 3:**
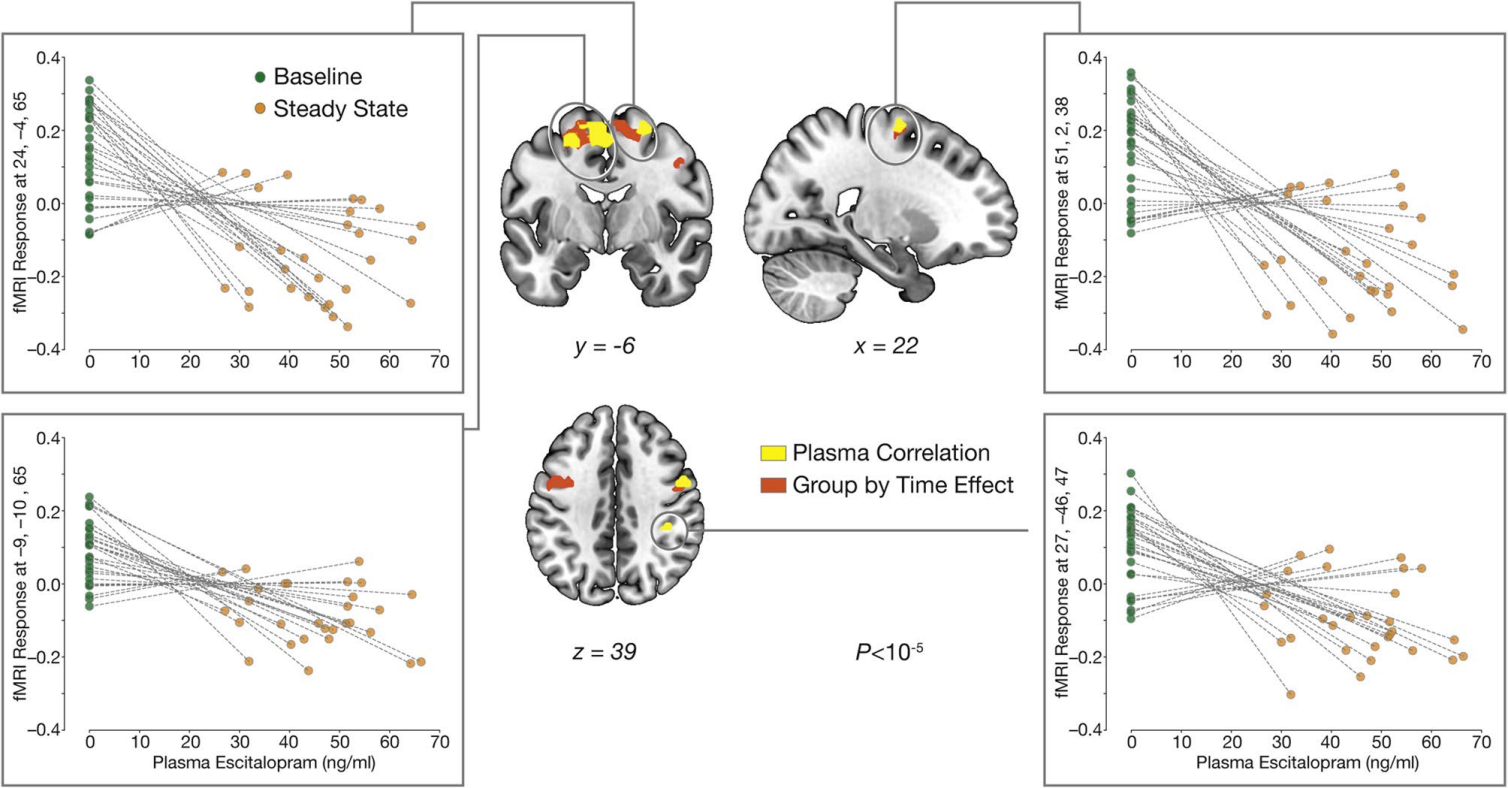
Correlation between decreased PPI Learning contrast and steady state escitalopram plasma kinetics. Inclusion of plasma escitalopram levels at both baseline and steady state as a covariate of interest shows a significant negative correlation between task dependent differences in functional thalamo-cortico connectivity in multiple motor regions (yellow). Results indicate a greater decrease in task-based thalamic connectivity with greater levels of escitalopram at steady state. Overlaid in red are the clusters from the significant group by time interaction (Fig. 1 - *Interaction*). All results are presented at a *p* < 10^–5^ cluster corrected threshold with a minimum cluster extent of 20 voxels. *BOLD* blood-oxygen-level-dependent.

Consideration of plasma escitalopram levels at baseline and at steady state yielded a significant negative correlation between the PPI Learning contrast and escitalopram plasma levels, with higher plasma escitalopram levels being associated with greater decreases in connectivity (Table 4). Extraction of the PPI Learning contrast estimates showed that higher plasma escitalopram levels are significantly associated with a lower PPI connectivity between the thalamus and the right superior frontal gyrus, the left SMA, the right M1, and the right superior parietal lobule (Fig. 3). Use of delta images (subtracting the PPI Learning contrast at steady state from baseline) yield a significant correlation between PPI connectivity change and steady state plasma escitalopram levels in bilateral cortical and subcortical regions (Supplemental Table 2; Supplemental Fig S1).

**Table 4:** Brain regions where decreases connectivity of the PPI Learning contrast from the thalamus correlates with increased plasma escitalopram levels.

Region	Cluster *p*(FWE_corr_)	Voxels	*t*-value	*z*-value	MNI (x,y,z)
R. superior frontal gyrus	0.007	23	6.74	5.16	24, − 4, 65
R. primary motor	0.017	35	6.34	4.95	51, 2, 38
R. superior parietal lobule	0.019	47	6.30	4.93	27, − 46, 47
			5.71	4.61	33, − 52, 59
			5.53	4.51	39, − 31, 41
L. supplementary motor area	0.024	133	6.19	4.87	− 9, − 10, 65
			6.12	4.84	− 39, − 13, 56
			5.83	4.56	− 24, − 10, 56

Results shown at *p* < 10^–5^ cluster forming threshold corrected with family-wise error (FWE) on the cluster level (≥ 20 voxels).

*L* left, *R* right, *peak MNI* Montréal Neurological Institute coordinates, *FEW* family-wise error.

Identification of high and low connectivity profiles within the escitalopram group, for each region observed in the correlation analysis, shows a significant interaction between baseline PPI connectivity and peripheral plasma escitalopram levels. This interaction shows that high baseline PPI connectivity is associated with a greater decrease at steady state (Fig. 3, Supplemental Tables 3–6).

### Associations between thalamo-cortico connectivity changes and behavioural outcome

Correlation analyses investigating a potential relationship between the PPI Learning contrast and mean sequence-specific behavioral outcomes do not yield a significant group difference when comparing escitalopram to placebo.

### Motion effects inside the MR scanner

Across groups and sessions, the mean framewise displacement (FD) was consistently below 0.36 mm. Less than 0.5% of frames from the entire study indicated single head movements by more than 1 mm. We did not observe any significant group differences in any FD motion parameter.

## Discussion

In this study, we employed PPI analysis to assess the effects of 1-week escitalopram-intake on functional brain connectivity during implicit sequence motor learning. By comparing a sequential Learning condition to a Simple motor learning condition (the PPI Learning contrast), our results show that, underlying a standard behavioral performance, functional connectivity from the thalamus to bilateral premotor and primary motor regions is significantly decreased in the Sequence Learning condition after 1-week of drug intake, compared to baseline. Additionally, we show that this decrease correlates with increases in escitalopram plasma levels between baseline and steady state, suggesting a parallel development between the degree of task-modulated connectivity decrease and the establishment of steady state escitalopram plasma levels. We did not observe any significant effect of escitalopram intake on PPI connectivity in any other seed region, relative to placebo. Moreover, we did not find any significant effect of escitalopram on any seed region in the PPI Motor contrast (i.e., comparing a combination of the Sequence and Simple Learning conditions to the Rest condition). Finally, we did not observe any significant change within the placebo group, with either the PPI Learning or PPI Motor contrasts.

Our main result indicates an escitalopram-induced decrease in thalamo-cortico PPI connectivity during implicit sequence motor learning. However, rather than a decrease of functional connectivity with the Sequence Learning condition, an alternative interpretation is an increase in connectivity associated with the Simple Learning condition. This seems less likely, however, given previous findings regarding escitalopram (and its racemic compound citalopram) on functional architecture, with observations of decreased connectivity in the presence of multiple doses^17,35,36^. Moreover, functional activity responses to sequence motor learning, particularly in premotor and parietal regions^37^, are hypothesized to constitute a decrease rather than an increase following training, a pattern that may extend to underlying connectivity. Considering our statistical model specification, we argue that our results represent a decrease in the Sequence Learning condition relative to the Simple Learning condition with respect to thalamo-cortico connectivity in response to escitalopram.

This decrease may reflect heightened automation of performance^38^, reduced neural energy consumption^39^, or increased independence between regions^40^ possibly via alterations in inhibitory tone, which has been proposed to occur in response to SSRIs^41^. Support for this explanation comes from findings of an inverse relationship between GABA and functional brain responses, both during motor learning^42^ and at rest^43,44^, with decreased fMRI response corresponding to increased GABA concentrations. An alternative explanation is the modulatory effect of escitalopram intake on inhibitory serotonin 1A receptor (5HT_1AR_) binding^45^, which itself is not only thought to be a mediator of subcortical neurogenesis^46,47^ but to have reciprocal interactions with action at the serotonin 2A receptor (5HT_2AR_) subtype^48^. Enhanced signaling of 5HT_2AR_ is hypothesized to lead to increased neural adaptability and plasticity, with acute pharmacological stimulation of these receptors leading to widespread decreases in functional network connectivity^49^, similar to the direction of change observed in this study. SSRI-induced modulation of receptor binding and action on GABAergic signaling could shift excitatory and inhibitory balance, thus allowing for improvements in neural automation of task performance, despite no difference in the behavioral performance between groups.

Complimentary to this interpretation is our observation of a negative correlation between connectivity change and peripheral plasma escitalopram levels. This finding indicates that higher plasma escitalopram levels at steady state are associated with greater decreases in thalamo-cortico PPI connectivity, particularly in participants with a higher baseline connectivity profile. While this variance could be attributable to trait differences in 5HT_1AR_ receptor density, which may determine baseline serotonin availability^50^, this finding may also be due to inherent differences in baseline connectivity. For example, baseline sensorimotor connectivity has been associated with variance in motor learning^51^ while whole-brain connectivity has been observed as a predictor of responses to SSRI intake^52^. This suggests that initial connectivity strength from the thalamus seed region may reflect baseline heterogeneity in individual network responsivity, thereby influencing the degree to which escitalopram exerts its neuromodulatory effects, possibly via a floor effect in participants with lower baseline connectivity. Future studies with larger sample sizes specifically designed to test this hypothesis are needed to clarify further.

Against our initial hypotheses, however, we did not observe any significant changes in PPI connectivity when using the M1, SMA, dlPFC, putamen, cerebellar or dPMC as seed regions. This may be due to a higher density of serotonergic binding sites in thalamic regions relative to the cortex^53^ and particularly, the cerebellum^54^. While transporter occupancy in the putamen and thalamus following SSRI-intake is comparable^55^, thalamic nuclei uniquely exhibit behaviorally relevant and reciprocal interactions with cortical motor regions, in which sustained bidirectional thalamo-cortico signaling modulates preparatory motor activity^56,57^. Moreover, these thalamo-cortico neural circuits operate on a cell-specific level, with excitatory loops, in particular, mediating this interaction^58,59^. This distinctive role of the thalamus as a central hub of motor-related neural circuitry^60^ may contribute to the specificity of our findings to this region. Additionally, we did not observe any significant changes in connectivity when comparing single dose with either baseline or steady state for any seed region. As prolonged SSRI-intake is required to downregulate auto-receptor feedback and to reduce serotonin reuptake^61^, early variance is 5HT_1AR_ responses to escitalopram may mitigate SSRI-induced alterations in PPI connectivity at single dose. Future studies with larger sample sizes may be needed to detect more subtle changes in PPI motor connectivity at early phases of SSRI-intake.

We also did not observe a significant group difference in a correlation between thalamo-cortico connectivity and the mean behavioral performance, an observation that is consistent with our behavioral findings which also did not show any difference in performance between groups. While this outcome could be a result of statistical power, or possibly a non-linear relationship between brain connectivity and behavioral outcomes, the use of an implicit sequence motor task may also be a contributor. Specifically, findings in unilateral stroke patients have shown that sequence learning undertaken with prior knowledge of sequences leads to improved performance when compared to extended practice of implicit learning alone^62^. This approach differs to the current study, in which participants implicitly learned the required sequence of pinch contractions and received no feedback regarding their performance. It is therefore possible that escitalopram may not modulate performance of implicitly-learned sequences, leading to a comparable learning curve between groups. In this case, the observed changes in thalamo-cortico connectivity may not necessarily correlate with this standard learning curve. Interestingly, we also did not observe any effect for the PPI Motor contrast. One possible explanation for this absence of evidence may be a possible loss of sensitivity resulting from the combination of the Sequence and Simple Learning conditions, which reflect different forms of motor performance. Moreover, the Rest condition was interleaved between motor conditions, possibly masking a “true” baseline, given the comparatively short duration of the condition. Specifically, this interleaved fashion could lead to a variable signal in Rest, possibly diminishing detectable effects of escitalopram when compared to this generalized motor condition. Studies with tasks with more explicit sequence learning and a more distinct Rest condition could provide more clarity on this question.

There are study limitations, however, that should be considered. First, we cannot rule out that our findings may be influenced by a difference in complexity between task conditions. Given that both the Sequence and Simple Learning conditions reflect motor learning, it is possible that the heightened difficulty associated with the Sequence Learning condition may draw on other domains not related to motor learning, such as attention, heightened visual perception, and general cognitive strategizing. The SPFT, however, was designed to exhibit a minimal learning effect for the Simple Learning condition, and recent preliminary findings have shown differentiated functional connectivity patterns when comparing these conditions at rest^63^, suggesting a distinction in the neural processing of each condition. Secondly, we also cannot rule out generalized SSRI effects that may be unrelated to the task. Even so, we employed an undirected modelling of task conditions^22^ and observed brain regions known to be involved in task processing (specifically, the bilateral premotor cortices). Additionally, task-independent thalamic connectivity has also been shown to be locally increased in response to escitalopram^18^, which differs to our observations during motor learning. Secondly, our sample consisted only of healthy female participants on oral contraceptives, making these findings difficult to generalize to male, naturally cycling female, healthy older, and stroke participants. However, this was a deliberate restriction to control for age, sex, pathology, and sex-hormone fluctuations on both escitalopram responsivity and motor learning. Third, we cannot comment on prolonged SSRI effects as intake took place across only 1-week. Future studies over several weeks are required to investigate long-term serotonergic modulation of PPI connectivity.

Fourth, we acknowledge that we have acquired a peripheral measure of escitalopram concentrations. As such, our findings of a relationship between PPI connectivity and plasma escitalopram levels should be interpreted with some caution. Future studies could address this with direct measures of the neural pharmacokinetics of SSRIs. Fifth, we acknowledge the relatively short training regimen and the early plateau in behavioral performance, which may limit the interpretability of these findings. This choice of 5 days, however, was made based on previous studies on sequence motor learning in health^26,29^ and in patients^20^ and to allow for escitalopram levels to reach a steady state plasma level, which is thought to occur after approximately 1-week of intake^64^. Finally, fMRI cannot assess the cellular effects of escitalopram on brain connectivity. Quantitative neuroimaging methodologies such as ^1^H MR spectroscopy estimates of glutamate and GABA, which have successfully imaged pharmacological interventions in thalamic regions and motor responses in cortical regions^65,66^ respectively, are needed to further discuss these interpretations.

In conclusion, we present results from the first study on the effects of 1-week SSRI-administration on functional connectivity during implicit sequence motor learning in health. While our results do not indicate an effect on behavioral performance, they do show that 1-week administration of escitalopram decreases thalamo-cortico connectivity when comparing two different levels of difficulty on a sequential motor learning task, and that these decreases are associated with peripheral levels of plasma escitalopram. As recent clinical trials in stroke patients assessed only behavioral responses to SSRIs, these findings highlight the importance of considering changes in fundamental brain architecture in response to combined sequence motor training and SSRI-intake. Given that motor learning is a viable model for post-stroke motor recovery^67^, our findings could further assist in advancing neural models of patient rehabilitation.

## Methods

### Sample

Participants were right-handed, 18–35 years old, with a body mass index (BMI) between 18.5 and 25 kg/m^2^, and female on oral contraceptives for ≥ 3 months (to control for sex differences in SSRI responsivity^68^, motor learning^13,69^, and sex-hormone effects on serotonin transporter density and serotonergic transmission^70^). Exclusion criteria were medication use, tobacco or alcohol intake, positive drug or pregnancy tests, professional musicianship or athleticism, an average of > 2 h video gaming per week, contraindications for MRI or SSRI-intake, and history of neurological or psychiatric illnesses. A physical examination, electrocardiogram recording, and hormonal analyses were performed prior to enrolment. Seventy-one participants enrolled, 60 of whom were included in analyses (escitalopram n = 29, placebo n = 31). During the experiment, 6 (escitalopram n = 4) participants voluntarily discontinued while data quality assessment excluded 2 participants (escitalopram = 1) due to head movement. Two further participants (escitalopram n = 1) were excluded due to MRI-related quality concerns. Finally, 1 placebo participant was excluded due to a pre-analytical error in plasma sample acquisition (Supplemental Fig S2). All participants provided written informed consent and received compensation.

### Design

**Figure 4:**
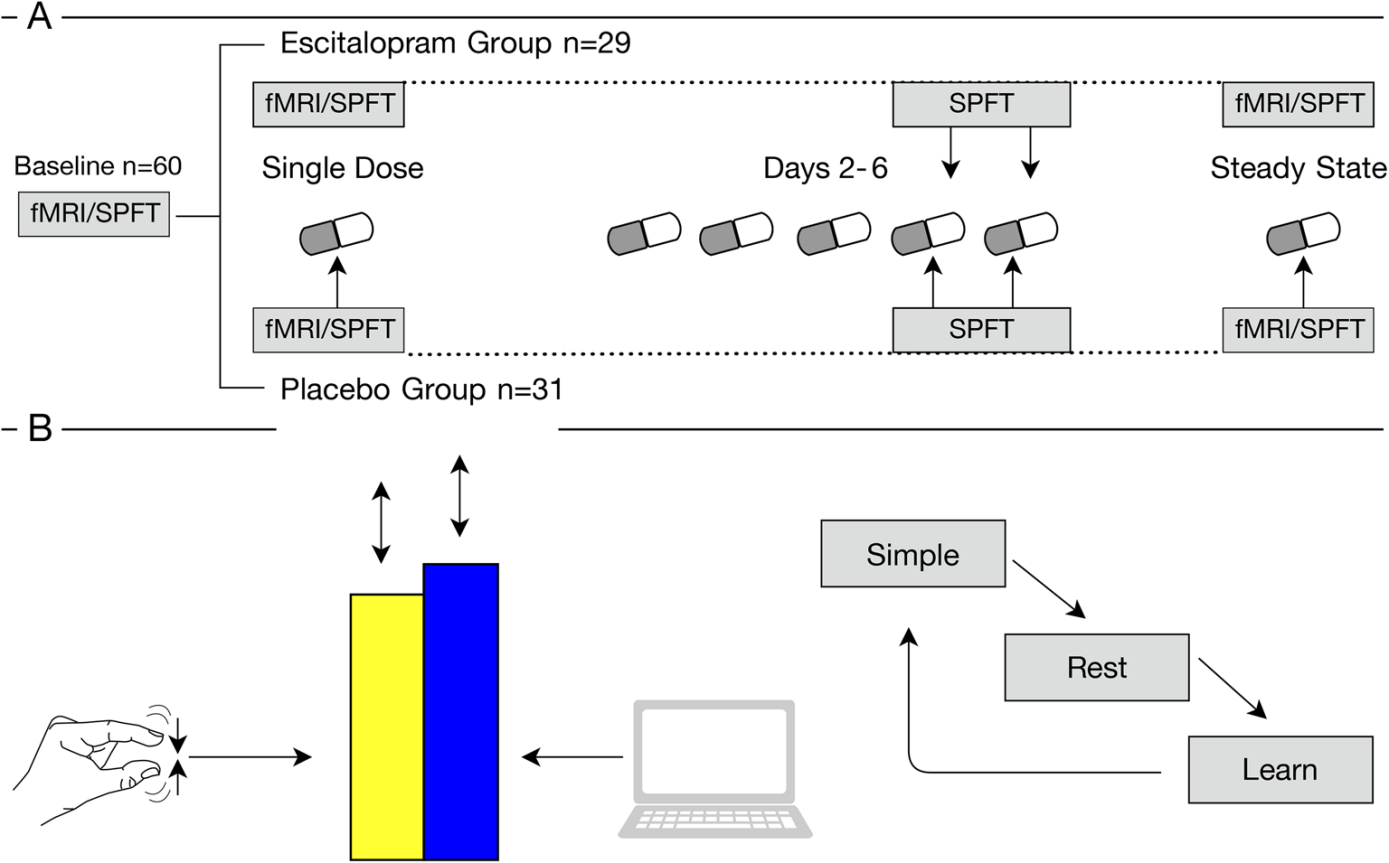
Study design and task stimulus. (**A**) Following a baseline fMRI and SPFT measurement, participants were randomized to receive either 20 mg of escitalopram or placebo for 7 days. Subsequent fMRI and SPFT measurements took place at both single dose and at steady state administration, with additional training sessions taking place on days 5 and 6 of administration. (**B**) Task stimuli consisted of a rising and falling computer-controlled blue bar. Participants, using their thumb and index finger to pinch the device controlling the yellow bar, had to match the rise and fall of the blue bar. Task conditions cycled through the Simple Learning (Simple), Rest, and Sequence Learning (Learn) conditions, with a total of 5 repetitions each. Each block consists of three trials. *fMRI* functional magnetic resonance imaging, *SPFT* sequential pinch force task.

The Ethics Committee of the Faculty of Medicine at Leipzig University approved all experimental procedures (approval number 390/16-ek), as governed by the Declaration of Helsinki, 2013. Seed regions were defined as a secondary outcome measure to a previously published study^14^. Following a baseline fMRI and motor learning measurement, participants were randomly assigned to orally receive either 20 mg escitalopram or placebo (mannitol/aerosol) from indistinguishable sequentially numbered containers, at fixed times, for seven consecutive days in a double-blind design. Task-fMRI was initially measured at baseline, prior to escitalopram or placebo intake. Following a baseline measurement, participants were randomized to either the escitalopram or placebo condition using an independent block randomization with a 1:1 allocation ratio, conducted by the Pharmacy of the University Clinic at Leipzig University. Task and fMRI measurements were subsequently performed after single dose escitalopram or placebo intake (single dose), and after 7 days of administration (steady state) (Fig. 4A). All measurements took place 3–4 h after intake, to allow for escitalopram to reach peak serum concentrations^64^. Both the experimenter and all participants were blind to group allocation.

### Sequence motor learning paradigm

We administered the sequential pinch force task (SPFT) during baseline, single dose, and steady state fMRI measurements with 2 additional behavioral assessments on days five and six of the experiment. Prior to entering the scanner, participants were verbally instructed on task requirements, which involved controlling the height of a yellow bar via the right-hand thumb and index finger, with stronger contractions leading to a greater increase in the bar’s height. Participants were asked to match their pinch strength to that of a computer controlled blue bar, the height of which rose and fell independently. No further verbal descriptions of either the Sequence Learning or Simple Learning conditions were given. Thus, participants were unaware of the specific requirements of differing task conditions and their interchanging onsets. Prior to the commencement of the measurements, participants performed an initial pinch, while positioned in the scanner. This served two purposes; to ensure participants understood task requirements and to act as an indicator of individual pinch strength. Task parameters were subsequently attenuated to account for this individual strength (measured in kg) to avoid variance associated with task difficulty. Motor learning was assessed with 2 conditions: a “Simple Learning” condition in which the blue bar moves sinusoidally, and a “Sequence Learning” condition in which the blue bar moves in a sequential pattern (stable across training sessions). Both conditions were prompted with on-screen text. A “Rest” condition, with no behavioral input in which the blue and yellow bars remained stationary, interspersed motor performance to avoid fatigue (Fig. 4B). Each task condition was repeated 5 times and participants received no feedback regarding performance.

### Analysis of screening variables

We used independent samples *t* tests using the R statistical programming language^71^ to assess potential group differences in age, BMI, and downregulated hormonal profiles. We quantified plasma escitalopram levels via a liquid chromatographic method^72^ using blood samples obtained at single dose and steady state from each participant.

### Analysis of sequential pinch force task performance

We used linear mixed effects modelling to analyze behavioral performance on the SPFT for the full sample (n = 60). To determine sequence-specific performance with regard to the Sequence Learning condition, we employed a linear model using the ‘lmer’ in R with the Lag (temporal deviation between the height of the computer-controlled and participant-controlled bars) as the outcome measure and time and condition (Sequence Learning/Simple Learning) as factors. We also applied a between-group linear modelling analysis, again with Lag as the outcome measure, and group and time as factors, to assess potential group differences in sequence motor learning behavior. A full description of these analyses and their results are reported elsewhere^14^.

### MRI data acquisition

We acquired fMRI data on a 3-Tesla MAGNETOM Verio scanner (Siemens Healthcare, Erlangen, Germany). We used a 32-element head coil and a gradient-echo Echo Planar Imaging (EPI) sequence (Flip angle 90°; repetition time (TR) 2 s; echo time (TE) 30 ms). A set of 30 axial slices (64 × 64 matrix, 3 × 3 × 3mm^3^ nominal resolution, with a field of view of 192 × 192 mm rotated − 15° away from the anterior to posterior commissure line to avoid prefrontal signal dropout) to acquire 495 volumes covering the whole brain (~ 16 min). Additionally, we acquired a whole-brain three dimensional T1-weighted Magnetization Prepared Rapid Gradient-echo (MPRAGE) with each functional measurement for image co-registration^73^ with inversion time (TI) 900 ms, TR 2300 ms, TE 2.98 ms, 1 × 1 × 1mm^3^ nominal isotropic resolution^74^ (~ 9 min).

### Functional MRI pre-processing and first level analysis

Data were pre-processed with Statistical Parametric Mapping (SPM12, v7771, implemented in MATLAB v9.7) and were realigned, slice-time corrected, co-registered, unwarped, normalized to Montréal Neurological Institute (MNI) space, and smoothed with a Gaussian kernel (8 mm full-width-half-maximum). Data processing included a high-pass filter of 1/128 Hz. Within the first level analysis, PPI contrasts were generated for each participant with the psycho-physiologic interaction module in SPM12. We used a general linear model (GLM) to specify two PPI contrasts: (1) the PPI Learning contrast comparing the difference in connectivity between the Sequence and the Simple Learning conditions whereby Sequence Learning connectivity patterns are greater than Simple Learning connectivity patterns. The Simple Learning condition, representing a simplistic contraction and release of the pinch device, was thus used to define a baseline of PPI connectivity to which sequence motor conditions could be compared. (2) The PPI Motor contrast, which combined the Sequence and the Simple Learning conditions into a single motor condition to compare general motor execution to the Rest condition.

**Figure 5:**
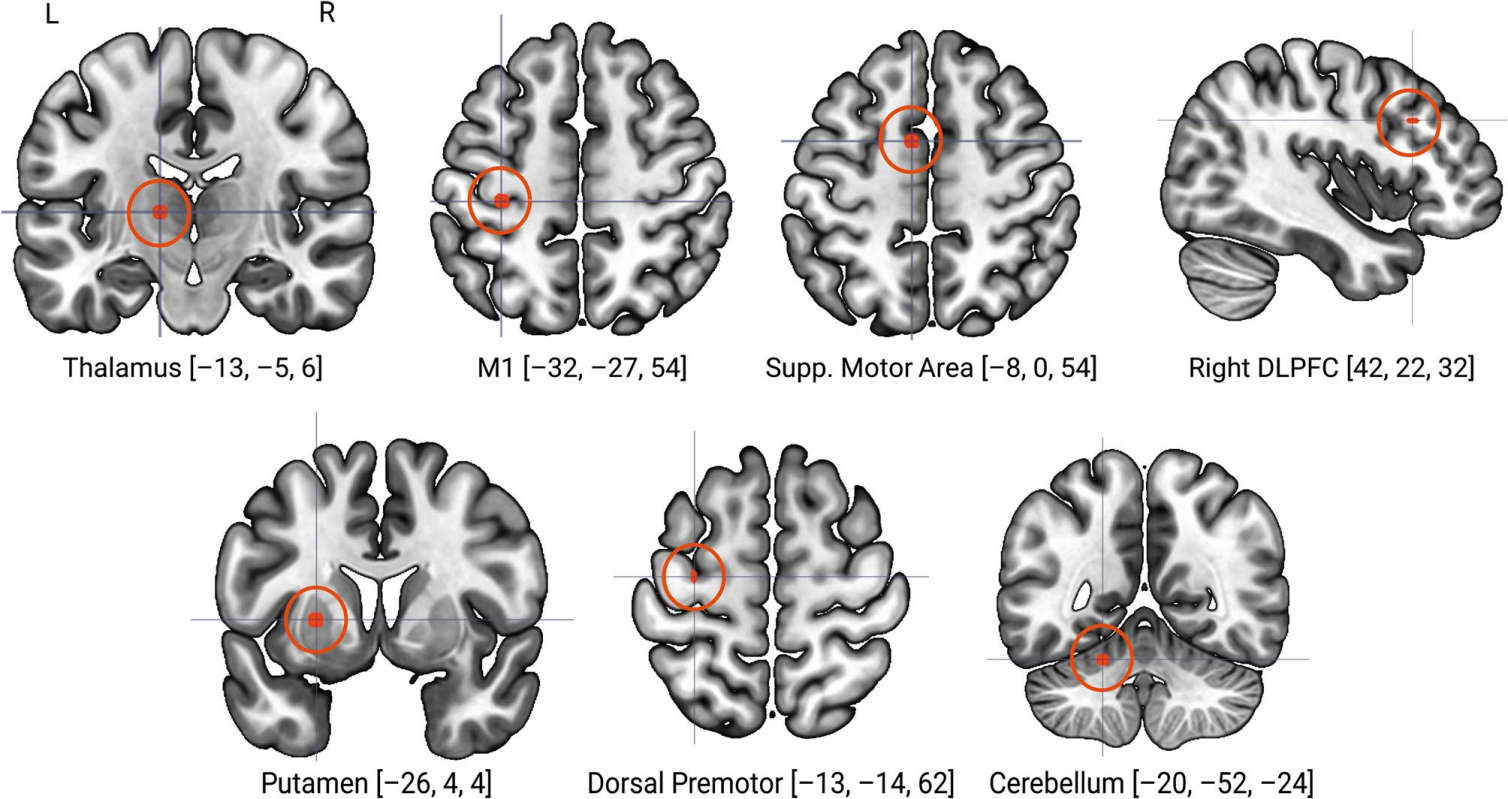
Psycho-physiological interaction seed regions. Orthogonal brain slices showing anatomical locations and corresponding Montréal Neurological Institute coordinates [x, y, z] for each PPI seed region, specified as a spherical region 3 mm in radius. All images show left to right orientation, while dlPFC shows a right sagittal view. *M1* primary motor cortex, *Supp. Motor Area* supplementary motor area, *DLPFC* Dorsolateral prefrontal cortex, *L* left, *R* right.

To specify the physiological component for each contrast, we selected the left hemisphere thalamus, primary motor cortex (M1), supplementary motor area (SMA), putamen, cerebellum, dorsal premotor cortex, and the right hemisphere dorsolateral prefrontal cortex (dlPFC) as 3 mm spherical seed regions of interest (Fig. 5). Using a *t*-contrast, we modelled the interaction between task conditions and the physiological neural time series by setting the psychological and physiological variables to zero and the interaction term to one. We approximated this interaction term with a deconvolution of the representative hemodynamic response function in order to normalize both psychological and physiological epochs to a uniform temporal resolution. All resulting contrast images for baseline, single dose and steady state were subsequently processed in second-level analyses.

### Second level analyses

Following PPI contrast specification, we employed a 2 × 2 flexible factorial model in SPM12 comparing group (escitalopram, placebo) changes in the PPI Learning contrast between: (1) baseline to single dose, (2) baseline to steady state, and (3) single dose to steady state, for each seed region. Within each model, we specified a ‘subject’ factor for pairwise analysis, a ‘time’ factor (time 1, time 2), and a ‘group’ factor (escitalopram, placebo). We specified *t*-contrasts to test for an interaction between the factors group and time, using both a positive and negative contrast to test for both directions of the interaction. Significant results were obtained using *p* < 0.05 family-wise error (FWE) corrected at the whole-brain voxel-level. In order to detect potential type II errors, we additionally employed an uncorrected threshold of *p* < 10^–5^ with a minimal extent threshold of 20 voxels. In addition, all interaction analyses that yielded a significant result were then validated with a non-parametric permutation analysis (5000 permutations) using the Threshold-free Cluster Enhancement (TFCE) toolbox in SPM12. All non-parametric tests were considered significant at a Bonferroni-corrected FWE α-threshold of *p* < 0.0012 (0.05/42—for the total number of bi-directional interaction contrasts employed across all seed seven regions and timepoints) to account for multiple testing. Only significant interactions replicated with non-parametric permutation tests were retained for post-hoc analyses.

### Post-hoc analyses

Post-hoc analyses for validated interaction analyses were conducted in a threefold fashion. Firstly, we assessed group-specific changes in the PPI Learning contrast with separate paired comparisons for each group. We compared changes in the PPI Learning contrast first within the escitalopram group and, in a separate analysis, within the placebo group. Secondly, we performed an independent samples *t* test in order to compare the escitalopram and the placebo group at baseline. Finally, we performed one-sample *t* tests to obtain the mean PPI Learning contrast for each group (escitalopram, placebo) at each time point (baseline, steady state), respectively.

### Extraction of contrast estimates

To investigate effect sizes of the PPI Learning contrast within regions showing a significant group × time interaction, we extracted beta values obtained by the parameter estimation from each significant region within the escitalopram group (n = 29). Additionally, we extracted betas from matching coordinates of the placebo group, for each of baseline, single dose, and steady state, in order to show effect size changes over the course of escitalopram administration, compared to placebo.

### Thalamo-cortico correlational analyses

We also assessed whether the observed changes in the thalamo-cortico PPI Learning contrast were associated with (1) plasma escitalopram levels within the escitalopram group and (2) sequence motor learning performance in the full sample. Here, we specified two separate flexible factorial designs, one for each analysis. In the first, plasma escitalopram levels were entered as a variable of interest^75^, while mean behavioral performance for each participant in the full sample were entered in the second. We assessed both directions for a potential correlation with thalamo-cortico connectivity. Analyses yielding a significant result were validated with a second approach by correlating baseline-steady state delta images (computed using the ‘fslmaths’ subtraction command) and entering these images in a third design to assess the correlation between the rate of change in thalamo-cortico connectivity and steady state plasma escitalopram levels.

### Interaction between baseline connectivity and plasma escitalopram levels

To assess individual variation in baseline connectivity on the association with plasma escitalopram levels, we performed an exploratory median split within the escitalopram group (n = 29) for each significant region observed in correlation analyses. Here, we specified two groups: high and low baseline connectivity, with one median split for each significant region. We specified an interaction term in SPM12 with the factors of profile (high, low) and the covariate plasma escitalopram levels, at baseline and steady state. With a *t*-contrast, we modelled the interaction between baseline connectivity profile and plasma escitalopram levels.

### Analysis of head motion inside the MR scanner

We assessed potential differences in head motion between groups and scans by assessing the framewise displacement (FD). Here, we used translational and rotational motion parameters obtained by SPM motion correction. All FD time courses were characterized by the mean and maximum FD, and the maximum FD after eliminating the largest 5% of the FD values, and the number of FD values exceeding 1 mm^76^.

### Ethical approval

The Ethics Committee of the Faculty of Medicine at Leipzig University approved all experimental procedures (approval number 390/16-ek). All methods were performed in accordance with the Declaration of Helsinki, 2013.

### Consent to participate

Written Informed consent was obtained from all individual participants enrolled in the study.

### Consent to publish

The authors affirm that human research participants provided informed consent for publication.

## Supplementary Information

Supplementary Information 1.
