# Supplementary material for: Decreased thalamo-cortico connectivity during an implicit sequence motor learning task and 7 days escitalopram intake
Source: Sci Rep. 2021 Jul 23;11:15060. doi: 10.1038/s41598-021-94009-7 (PMC8302647; doi:10.1038/s41598-021-94009-7)
Supplement: Supplementary file 1 — Supplementary Information 1. [file 41598_2021_94009_MOESM1_ESM.docx]

**Supplemental Information**

**Decreased thalamo-cortico connectivity during an implicit sequence motor learning task and seven days escitalopram intake**

Eóin N. Molloy, Rachel G. Zsido, Fabian A. Piecha, Nathalie Beinhölzl, Ulrike Scharrer, Gergana Zheleva, Ralf Regenthal, Bernhard Sehm, Vadim V. Nikulin, Harald E. Möller, Arno Villringer, Julia Sacher¶ & Karsten Mueller¶.

¶ Equal Contribution

Supplemental Tables 1-6

Supplemental Figures S1-S3

**Supplemental Table 1.** Mixed effects modelling of sequence motor and simple control task conditions in the full sample (n=60). Results show omnibus effects of time and task condition (Learning/Simple), without regard to group (escitalopram/placebo), with corresponding *p*-value and associated effect sizes. LRT = likelihood ratio test, df = degrees of freedom, χ2 = Chi-square. *significant contribution to model.

| Mixed Effects  Modelling (Lag) | Fixed Effects | LRT | | Marginal *R*^2^ | Conditional *R^2^* |
| --- | --- | --- | --- | --- | --- |
|  |  | χ^2^ (df) | *p*-value |  |  |
| Intercept | - | - | - | 0 | 0.212 |
| Time | time | 1127.5 (24) | < 0.001* | 0.089 | 0.304 |
| Condition | condition+time | 4641.4 (1) | <0.001* (Lag_Time) | 0.338 | 0.547 |
| Interaction | condition*time | 2992.0 (24) | < 0.001* | 0.458 | 0.659 |

**Supplemental Table 2**.Brain regions showing a significant association with steady state plasma escitalopram levels. Inclusion of delta images in a one-sample *t*-test with steady state plasma levels as a covariate of interest reveal widespread correlations between PPI connectivity changes and plasma escitalopram levels. Results are shown at a standard *p*<0.001 cluster forming threshold corrected for multiple comparisons with family wise error (*p*<0.05) on the cluster level. L=left, R=right, cluster MNI=Montréal Neurological Institute coordinates. FWE=Family-wise error.

| Region | Cluster *p*(FWE-corr) | | Voxels | *t-*value | *z*-value | MNI (x,y,z) |
| --- | --- | --- | --- | --- | --- | --- |
| R. Superior Temporal Gyrus | <0.001 | 156 | | 5.95 | 4.75 | 63, -31, 14 |
|  |  |  | | 4.83 | 4.09 | 57, -19, 35 |
| R. Precentral Gyrus | <0.001 | 134 | | 5.85 | 4.69 | 54, 2, 35 |
|  |  |  | | 3.85 | 3.42 | 51, -1, 53 |
|  |  |  | | 3.78 | 3.37 | 54, -7, 47 |
| L. Supramarginal Gyrus | <0.001 | 1547 | | 5.83 | 4.68 | -51, -25, 35 |
|  |  |  | | 5.51 | 4.5 | -57, -19, 38 |
|  |  |  | | 5.44 | 4.46 | -48, -16, 53 |
| L. Middle Occipital Gyrus | 0.002 | 94 | | 5.38 | 4.42 | -51, -73, -1 |
|  |  |  | | 4.84 | 4.09 | -36, -88, -4 |
|  |  |  | | 4.11 | 3.61 | -42, -79, -4 |
| L. Precentral Gyrus | 0.001 | 119 | | 4.96 | 4.17 | -57, 5, 20 |
|  |  |  | | 4.84 | 4.09 | -51, 2, 38 |
| R. Superior Parietal Lobule | <0.001 | 253 | | 4.79 | 4.06 | 27, -46, 47 |
|  |  |  | | 4.53 | 3.89 | 24, -52, 62 |
|  |  |  | | 4.31 | 3.74 | 33, -52, 59 |
| L. Cerebellum | 0.001 | 109 | | 4.71 | 4.01 | -45, -55, -34 |
|  |  |  | | 4.48 | 3.86 | -24, -70, -22 |
|  |  |  | | 4.36 | 3.77 | -36, -46, -25 |
| R. Thalamus | 0.004 | 85 | | 4.67 | 3.98 | 15, -25, 14 |
|  |  |  | | 4.21 | 3.67 | 21, -31, 11 |
|  |  |  | | 3.91 | 3.46 | 15 -13, 20 |
| L. Thalamus | 0.007 | 78 | | 4.57 | 3.91 | -15, -37, 11 |
|  |  |  | | 3.89 | 3.45 | -30, -40, -4 |
|  |  |  | | 3.86 | 3.43 | -12, -22, 14 |

**Supplemental Table 3.** Brain regions showing a significant interaction between baseline connectivity profile in the right hemisphere superior frontal gyrus (high vs low) and steady state plasma escitalopram levels. Results are shown at a *p*<10^-5^ cluster forming threshold with a minimal voxel extent of 20, corrected for multiple comparisons with family wise error (*p*<0.05) on the cluster level. L=left, R=right, cluster MNI=Montréal Neurological Institute coordinates. FWE=Family-wise error.

| Region | Cluster p(FWE-corr) | Voxels | *t*-value | *z-*value | MNI (x,y,z) |
| --- | --- | --- | --- | --- | --- |
| L. Posterior Med. Frontal Cortex | <0.001 | 164 | 8.64 | 5.93 | -3, 11, 59 |
|  |  |  | 6.66 | 5.08 | -6, 14, 47 |
|  |  |  | 6.31 | 4.9 | 9, 2, 59 |
| R. Superior Frontal Gyrus | <0.001 | 79 | 7.39 | 5.42 | 24, -7, 62 |
|  |  |  | 6.28 | 4.89 | 9, -16, 65 |
|  |  |  | 5.29 | 4.35 | 12, -1, 68 |
| L. Primary Motor | <0.001 | 27 | 6.51 | 5.00 | -36, -7, 56 |

**Supplemental Table 4.** Brain regions showing a significant interaction between baseline connectivity profile in the right hemisphere primary motor cortex (high vs low) and steady state plasma escitalopram levels. Results are shown at a *p*<10^-5^ cluster forming threshold with a minimal voxel extent of 20, corrected for multiple comparisons with family wise error (*p*<0.05) on the cluster level. L=left, R=right, cluster MNI=Montréal Neurological Institute coordinates. FWE=Family-wise error.

| Region | Cluster p(FWE-corr) | Voxels | *t*-value | *z-*value | MNI (x,y,z) |
| --- | --- | --- | --- | --- | --- |
| L. Inferior Parietal Lobule | <0.001 | 106 | 7.39 | 5.42 | -36, -46, 44 |
|  |  |  | 6.66 | 5.08 | -48, -43, 47 |
| R. Primary Motor | <0.001 | 49 | 6.75 | 5.12 | 45, 2, 41 |
| R. Posterior Med. Frontal | <0.001 | 27 | 6.38 | 4.94 | 9, 11, 53 |
|  |  |  | 5.64 | 4.55 | 3, 20, 47 |
| R. Insula | 0.001 | 23 | 6.13 | 4.81 | 33, 23, 8 |

**Supplemental Table 5.** Brain regions showing a significant interaction between baseline connectivity profile in the right hemisphere superior parietal lobule (high vs low) and steady state plasma escitalopram levels. Results are shown at a *p*<10^-5^ cluster forming threshold with a minimal voxel extent of 20, corrected for multiple comparisons with family wise error (*p*<0.05) on the cluster level. L=left, R=right, cluster MNI=Montréal Neurological Institute coordinates. FWE=Family-wise error.

| Region | Cluster  p(FWE-corr) | Voxels | *t*-values | *z-values* | MNI (x,y,z) |
| --- | --- | --- | --- | --- | --- |
| R. Precuneus | <0.001 | 137 | 8.42 | 5.85 | 27, -46, 47 |
|  |  |  | 7.52 | 5.48 | 33, -43, 41 |
|  |  |  | 6.25 | 4.87 | 48, -34, 44 |
| L. Inferior Parietal Lobule | <0.001 | 137 | 8.24 | 5.78 | -33, -43, 41 |
|  |  |  | 6.63 | 5.06 | -51, -40, 50 |
|  |  |  | 5.83 | 4.65 | -51, -28, 41 |
| R. Posterior Med. Frontal Cortex | <0.001 | 78 | 6.76 | 5.13 | 9, 5, 53 |
|  |  |  | 6.11 | 4.80 | -3, -13, 56 |
| L. Primary Motor | <0.001 | 31 | 6.34 | 4.92 | -27, -10, 56 |
| L. Cerebellum | 0.001 | 23 | 5.87 | 4.67 | -3, -67, -13 |

**Supplemental Table 6.** Brain regions showing a significant interaction between baseline connectivity profile in the left hemisphere supplementary motor area (high vs low) and steady state plasma escitalopram levels. Results are shown at a *p*<10^-5^ cluster forming threshold with a minimal voxel extent of 20, corrected for multiple comparisons with family wise error (*p*<0.05) on the cluster level. L=left, R=right, cluster MNI=Montréal Neurological Institute coordinates. FWE=Family-wise error.

| Region | Cluster  p(FWE-corr) | Voxels | *t*-values | *z*-values | MNI (x,y,z) |
| --- | --- | --- | --- | --- | --- |
| L. Posterior Med. Frontal Cortex | <0.001 | 260 | 9.1 | 6.1 | -9, -10, 65 |
|  |  |  | 6.69 | 5.09 | -48, -19, 50 |
|  |  |  | 6.52 | 5.01 | -24, -7, 59 |
| L. Inferior Parietal Lobule | <0.001 | 24 | 6.52 | 5.01 | -36, -49, 47 |

**
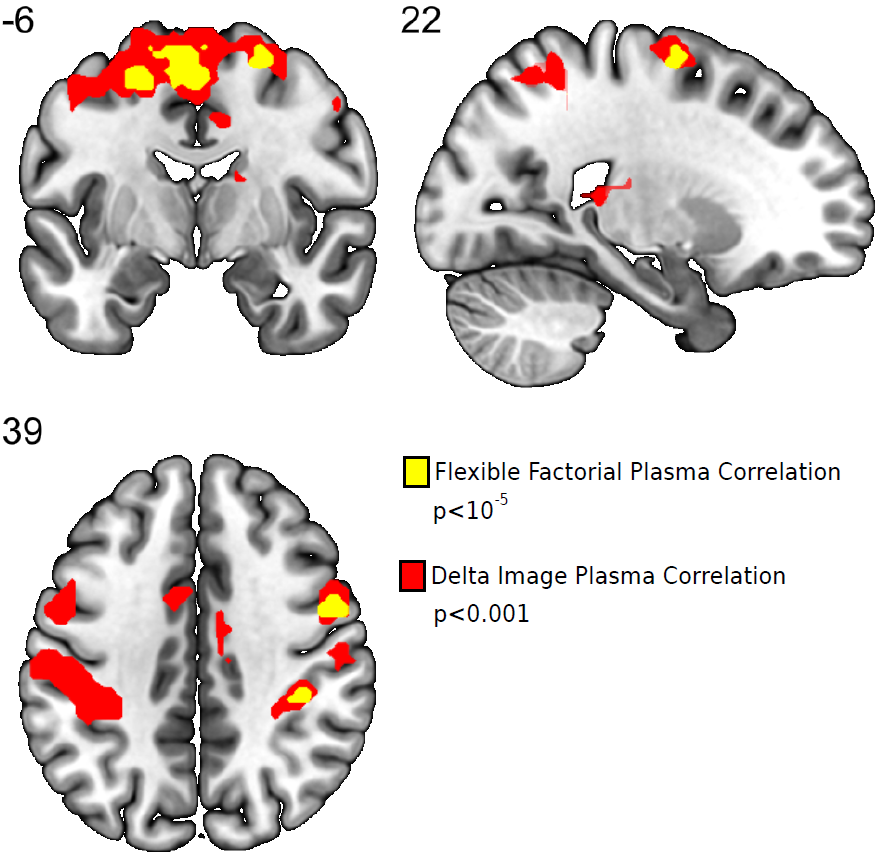
**

**Supplemental Figure S1.** **Comparison of flexible factorial vs one-sample *t*-test approach for PPI connectivity and plasma escitalopram correlational analysis.** Correlational analyses were conducted in two separate approaches; (i) a flexible factorial design to assess absolute changes from baseline to steady state in thalamic PPI connectivity and plasma escitalopram levels (yellow) and (ii) using delta images (i.e., rate of change) from baseline to steady state with a single image for each escitalopram participant correlated with steady state plasma escitalopram levels (red). Results from each approach are consistent showing significant effects in multiple bilateral brain regions. Flexible factorial results are presented at a p<10^-5^ threshold while results from the delta approach are presented at an exploratory p<0.001 cluster forming threshold.

**
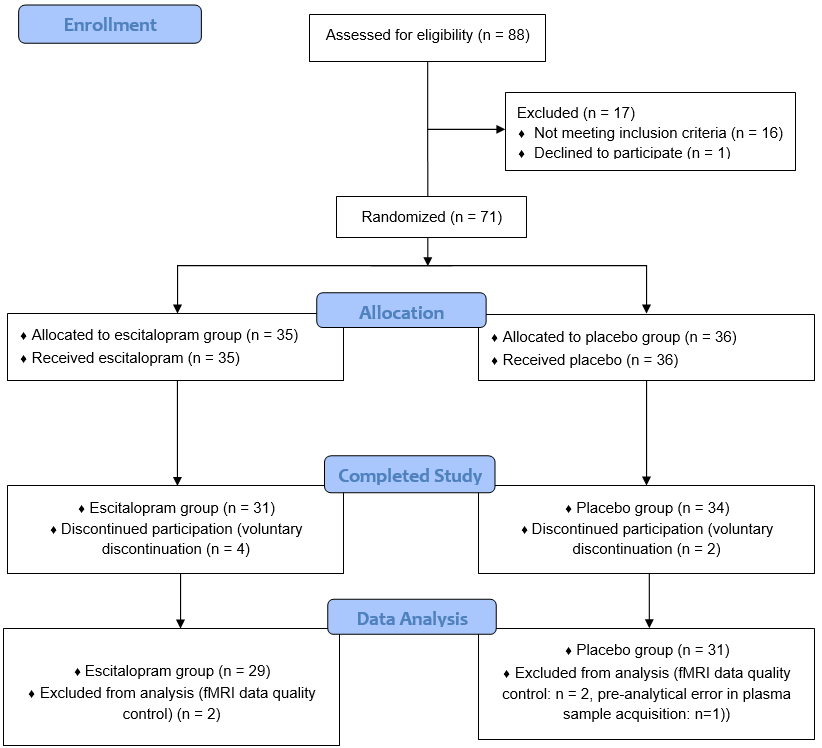
**

**Supplemental Figure S2.** **Flowchart detailing the screening, inclusion, randomization, and exclusion procedure**. Six participants chose to voluntarily discontinue participation (Placebo = 2/escitalopram = 4). Two participants who completed the study (Placebo = 1, escitalopram = 1) were excluded due to excess head movement as calculated by framewise displacement (>3SD outside the mean), the presence of structural (placebo n=1) and functional (escitalopram n=1) image artifacts, and a pre-analytical error in plasma acquisition (placebo n=1).


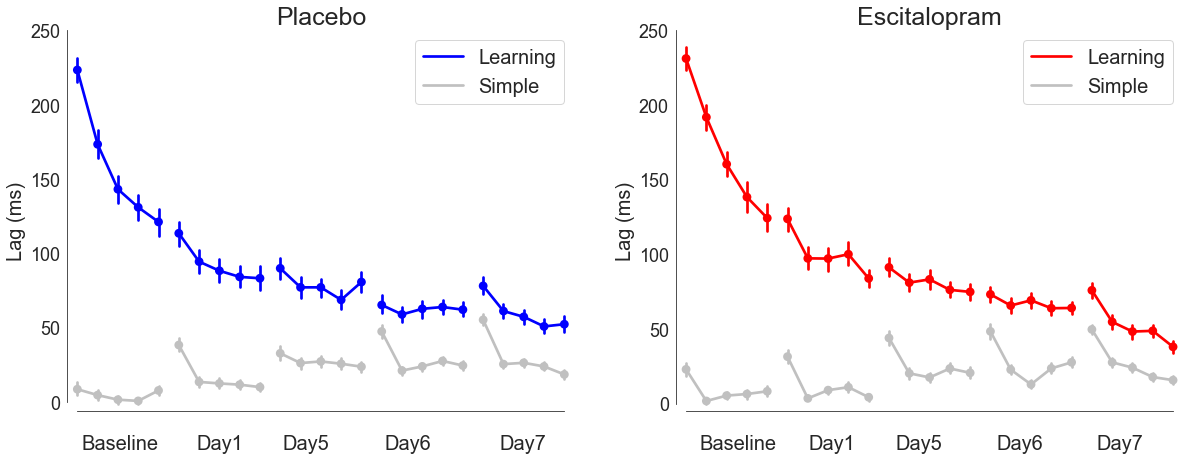


**Supplemental Figure S3. Differential performance between the sequence-specific motor learning condition and the simple control condition for all participants (n=60).** Results show a statistically significant difference between Lag performance (the temporal deviation between the computer controlled and participant controlled bars) on the sequence-specific motor condition (Learning) and the motor execution control condition (Simple). Performance for the placebo group (left) and the escitalopram group (right) did not differ significantly.
